# Geospatial analysis and scale-up modelling of the impact of mobile programming on access to essential childhood vaccinations in Yemen

**DOI:** 10.1038/s43856-025-00762-5

**Published:** 2025-04-18

**Authors:** Kent Garber, Priyanka Kanth, Kebir Hassen, Sherin Varkey, Kyandindi Sumaili

**Affiliations:** 1 University of California, San Francisco, San Francisco, CA USA; 2 World Bank, Washington, DC USA; 3 UNICEF, Sana’a, Yemen; 4 World Bank, Addis Ababa, Ethiopia

**Keywords:** Health services, Public health

## Abstract

**Background:**

Childhood vaccination rates in Yemen remain alarmingly low, and recent political developments have restricted vaccine delivery. Understanding population access to vaccinations through existing delivery modalities, including fixed and mobile services, is critical to inform strategies to improve access, increase coverage, and prevent outbreaks.

**Methods:**

We developed a geospatial model of national and subnational vaccination access via fixed health facilities and mobile health teams, using newly developed datasets accounting for conflict-related effects. We calculated travel times to fixed health facilities and mobile sites offering immunizations and estimated the number and percentage of the under-5 population with access to vaccines. We also conducted a scale-up model to identify and prioritize locations where additional mobile teams would most improve vaccination access.

**Results:**

We estimate that in 2023, 66.3% (3.57 million) of the Yemeni under-5 population lived within a 30-minute walk of a health facility or mobile site offering vaccinations, compared to 56.5% living within a 30-minute walk of a health facility alone. Even with mobile contributions, we find substantial regional variation; in 13 of 22 governorates, more than one-third of children cannot access a vaccination site within 30 min. In our scale-up analysis, we identify optimal locations for 300 new mobile sites that would increase access by an additional 7.2% of the population.

**Conclusions:**

Using mobile teams improves geographic access to vaccinations in Yemen; however, significant access gaps will remain even with dedicated scale-up efforts. Geospatial modelling offers a rigorous approach to evaluating the impact of different service delivery methods in conflict settings and can help optimize targeting strategies.

## Abstract (web summary)

Garber et al examine the impact of mobile programming on childhood vaccination access in Yemen using geospatial modelling and conflict-adjusted datasets. The modelling also identifies a set of optimal locations for new vaccination sites and quantifies their potential impact on vaccine accessibility.

## Plain language summary

Low childhood vaccination coverage is a major challenge in conflict-affected countries, including Yemen. Mobile vaccination teams have become a key strategy to improve vaccine uptake, but it remains unclear whether they make it easier for people to access vaccinations. We assessed the national and sub-national impact of mobile services on vaccination access in Yemen and identified optimal locations for expansion. Our findings show that mobile services enhance geographic access to childhood vaccinations, particularly for hard-to-reach populations. Additionally, scaling up services in targeted locations could further improve access, though some gaps would remain. These insights could inform national strategies to enhance vaccination coverage and broader primary healthcare access in Yemen and be adapted for use in other countries.

## Introduction

Gaps in health services are common in conflict-affected settings. Health infrastructure damages, supply chain interruptions, and health worker displacement frequently exacerbate pre-existing health system weaknesses, impairing health service delivery^1^. At the same time, populations often face substantial barriers (e.g., insecurity, road closures, lack of transportation, lost income) accessing health facilities. These obstacles have profound implications for health outcomes and can contribute to declines in health service coverage^2,3^.

Reflecting these realities, alternative approaches to health service delivery such as mobile programming are increasingly popular in humanitarian and protracted conflict settings. Over the past decade, UN agencies, non-governmental organizations, and other stakeholders have deployed mobile clinics in many of the world’s most prominent conflicts, including Iraq, Syria, Afghanistan, South Sudan, and Ukraine^4–8^. Depending upon the context, these teams may operate independently, in an ad hoc or time-limited fashion, or as extensions of fixed healthcare facilities run by public authorities^9–11^. Their geographic scope can range from a few settlements to regional or national coverage. Their frequency of visits and range of services (e.g., vaccinations, nutrition, maternal and child health services, etc.) may also vary widely.

Despite widespread use, the literature on mobile clinic implementation in conflict spaces is limited, and few studies have directly examined their impact. A 2020 review found only five studies that formally evaluated mobile clinics in conflict-affected areas: two in Haiti, and one each in Afghanistan, the Democratic Republic of Congo, and the Occupied Palestinian Territories^4,12–16^. Metrics for evaluation (e.g., appropriateness, coverage, effectiveness, efficiency) varied across studies, and although some studies reported positive results, the review concluded that a stronger evidence base was needed to guide decision-making around their design and implementation. Additionally, we identified only one peer-reviewed study (from Afghanistan) that examined mobile health units deployed as part of a longer-term national health system strategy in a conflict-affected country^11^.

In Yemen, which has faced protracted internationalized armed conflict since 2014, mobile teams have long been an important part of the country’s vaccination delivery programme and the international humanitarian response. For more than a decade, Yemen public health officials and stakeholders have implemented a multi-pronged strategy to deliver childhood vaccinations, including (1) offering vaccines at fixed, publicly supported health facilities; (2) supporting integrated outreach rounds, conducted several times a year, in locations 30–90 min from a fixed health facility; and (3) deploying mobile teams to reach more distant communities. Governorate and district health offices typically select the localities to receive mobile clinic support, with priority given to remote villages with low service coverage based on local knowledge and microplanning exercises. Mobile teams offer essential childhood vaccines (including pentavalent, measles, rubella, and polio immunizations), as well as basic nutrition and reproductive health services. The teams comprise multiple health workers, including one doctor, one vaccination specialist, one reproductive health specialist, and two nutrition workers. Teams visit the selected localities approximately once every two weeks, according to a schedule approved and advertised by community leaders, who also select the physical location where the mobile clinics work. These may include mosques, schools, homes of religious or political leaders, or open spaces designated by local authorities.

Despite these efforts, vaccination rates in Yemen remain concerningly low, reflecting the toll of more than nine years of conflict, political instability, economic challenges, and other factors. As of 2023, fewer than 55% of children were fully covered against diphtheria, pertussis, and tetanus, and fewer than half were vaccinated against measles^17^. Under-5 mortality rates in Yemen have stagnated and remain the highest in the Middle East and North Africa (MENA) region^18^. Several deadly measles and polio outbreaks have recently occurred, with more than 270 polio cases documented since 2021. Political developments have further impaired large-scale vaccination efforts^19^. In 2023, the Ministry of Public Health and Population (MOPHP) in the northern governorates suspended many vaccination activities outside of fixed health facilities. This included a complete stop to integrated outreach activities that accounted for up to 30% of the annual immunization coverage. Meanwhile, only 55% of Yemen’s health facilities are fully functional, and more than 30% do not offer immunization services^20^. Moreover, travel permit delays and movement restrictions have further complicated service delivery efforts.

Mobile health teams, by contrast, have mostly been allowed to continue operating. As a result, donors and UN agencies have undertaken a concerted effort to optimize their deployment to address worsening service delivery gaps and preserve vaccination coverage amid the shifting political landscape. This optimization effort has highlighted the need to better understand where mobile health teams are working, how they contribute to vaccination access, and which areas need additional support.

Geospatial analyses can be a particularly useful tool for policy planners and implementing agencies facing questions around resource deployment and healthcare access. In recent years, several studies have used geospatial techniques to optimize the deployment of community health workers, scale-up of health posts in rural areas, and improve access to emergency obstetric services in low-and-middle income countries^21–24^. A growing body of open-source datasets and advances in geospatial information systems (GIS) have allowed researchers to develop sophisticated regional and global models of healthcare access^25,26^. Conflict settings introduce specific challenges for geospatial modelling, due to the impact of conflict on health facility functionality, service availability, population distribution, and road networks^27^. Despite these challenges, prior research has demonstrated that geospatial modelling, informed by updated, context-specific inputs, can inform decision-making in these contexts^28^.

Here we present the results of a geospatial modelling study using what are, to our knowledge, novel, conflict-adjusted datasets to assess the current contribution of mobile teams to childhood vaccination access in Yemen. This analysis quantifies the additive effect of mobile services on vaccination access in a protracted humanitarian setting using updated datasets that account for internal displacement, road access restrictions, and other movement barriers, an approach that we have not previously seen documented in the literature. Additionally, through a geospatial optimization analysis, we identify locations where populations lacking access to vaccinations would benefit the most from further scale-up of vaccine delivery sites. The findings are immediately relevant for country stakeholders’ ongoing efforts to reduce the number of unvaccinated children, respond to political realities on the ground, and prevent future disease outbreaks.

## Methodology

A series of geospatial modelling analyses were performed to examine geographic access to Extended Programme for Immunization (EPI) services at fixed health facilities and mobile clinic sites in Yemen in 2023 and to explore the impact of potential scale-up scenarios on future EPI access.

### Study area

Accessibility to vaccination services was calculated for all of Yemen, which has an estimated 2023 population of 34.7 million with 5.4 million children under the age of 5, as well as each of its 22 governorates (the first-level administrative region, Supplementary Fig. 1). Yemen is further sub-divided into 333 districts and 2149 subdistricts, the latter of which was used in the scale-up analysis.

### Data Sources

Data on health facility type, location, functionality status, and availability of EPI services at fixed sites were obtained from the Yemen Health Resources and Service Availability Monitoring (HeRAMS) survey, completed in 2023^20^. HeRAMS is a standardized data collection instrument developed by the World Health Organization (WHO) specifically for use in fragile and emergency contexts. Designed to provide a timely, comprehensive census of publicly operated health facilities in contexts where data are frequently outdated and health information systems are weak, HERAMS captures data on facility geolocation, functionality, and service availability, including EPI services. In Yemen, WHO worked with the Ministry of Public Health and Population in all 22 governorates to train monitors, collect data through monitored facility visits, and verify findings as part of the HeRAMS process. Data were shared by WHO for use in this analysis. Geocoordinates of the locations of mobile health teams providing EPI services were provided by the UNICEF Yemen country office.

Road network data was initially obtained from Open Street Map, an open-source global collaborative that provides detailed mapping and classification of road networks^29^. Given that many roads in Yemen have been affected by the conflict, we obtained updated datasets from the UN Yemen Logistics Cluster, reflecting closed and “difficult to access” roads, as of December 2023^30^. These datasets were georeferenced in QGIS, and the Open Street Map road network was modified by removing closed road segments and separately tagging “difficult to access” segments. Additionally, all road types were reclassified as primary, secondary, and tertiary roads for incorporation into the geospatial model, similar to other published geospatial analyses^24^.

Elevation data were obtained from the Shuttle Radar Topography Mission (SRTM) digital elevation model (DEM), an international project led by the National Aeronautics and Space Administration (NASA) and the National Geospatial-Intelligence Agency (NGA)^31^.

Land cover data were obtained from the ArcGIS Living Atlas repository and the Sentinel-2 Land Cover Explorer at the 10 m x 10 m resolution and resampled at the 90 m x 90 m level to match the elevation data resolution^32^. The satellite metadata classify land type according to a set of internationally standardized categories (e.g., water, trees, flooded vegetation, crops, built area, bare ground, snow/ice, clouds, and rangeland). All landcover categories present in the Yemen satellite data were retained in the geospatial analysis.

Administrative data on governorate, district, and sub-district boundaries were obtained from the Humanitarian Data Exchange, an online data repository updated and maintained by humanitarian and development stakeholders^33^.

Finally, new spatially gridded estimates of the 2023–2024 Yemen population were commissioned through a collaboration between the World Bank and WorldPop, a non-profit organization specializing in producing high-resolution spatially distributed population estimates in low and middle-income countries. The new Yemen estimates used in this analysis were produced using updated population modelling from the UN Office for the Coordination of Humanitarian Affairs (UN OCHA) in Yemen, satellite data, and prior 2004 census data using a validated methodology previously described elsewhere^34–36^. The UN OCHA estimates were developed with the Yemen Central Statistical Office and account for population growth as well as internally displaced persons (IDPs) through 2023 at the district level. As these estimates are disaggregated by age group in five-year increments, WorldPop developed multiple population spatial raster datasets, including one of the full population and one of the age 0–4 population. The latter is used in this analysis as it most closely represents the target population for childhood vaccinations. Raster datasets were produced at the 100 m x 100 m level of spatial resolution using a constrained methodology, in line with standard practices for geospatial modelling of this type.

No IRB approval was required because the study did not involve human subjects and did not collect or analyze identifiable private information.

### Main outcome measures

For all analyses, geographic access to EPI services was defined primarily as a 30-minute walk time to the nearest health facility or mobile team site offering EPI. This definition is based on two context-specific realities. First, under Yemen’s long-standing vaccination strategy, fixed facilities are expected to serve populations within a 30-minute travel time, whereas outreach and mobile services have historically targeted more remote populations. Second, UNICEF programmatic surveys show that the majority of Yemeni beneficiaries (>70%) who access primary care services at fixed facilities report walking 30 min or less and do not use motorized transport. For these reasons, we use 30-minute walk times as our primary definition of access. However, we also report accessibility across a range of time thresholds and travel parameters, as described in more detail below.

### Data analysis

A geospatial model of EPI accessibility in Yemen was developed using AccessMod 5, incorporating elevation, land cover, road network, service availability, and spatially distributed population data. AccessMod uses a cost path distance algorithm to calculate access, first producing a friction raster surface based upon integrated land cover, elevation, and road network data, then incorporating facility (or mobile site) locations and travel speeds to derive estimates of population coverage^23,24,37^. The modelling generates accessibility maps in raster format, representing travel times in each grid-cell to the nearest EPI site, as well as tabular data on population coverage at the national and subnational level derived through zonal statistics.

We ran multiple analyses to estimate geographic access to vaccinations through currently available vaccination modalities. First, we ran a ‘fixed health facility only” analysis, calculating the number of children under age 5 within a continuum of walking times (ranging from <10 min to >4 h) to the nearest fixed health facility offering EPI services. Second, we ran a “fixed health facility plus mobile clinic” analysis, i.e. calculating the number of people within the same continuum of walking times to a fixed facility or mobile health site offering EPI services. For these analyses, we focus primarily on the 30-minute walk time outputs, given the importance of the 30-minute parameter in national vaccination delivery planning. However, we also show the results for other time thresholds to illustrate their varying effect on access. The difference between the outputs of the fixed-only and fixed-and-mobile analysis was then calculated to isolate the additive contribution of mobile sites to immunization access.

Travel scenarios were informed by inputs from UNICEF local staff in Sana’a and Aden, as well as published studies^24^. For the walking-only models, travel speeds were determined based upon the type of land cover being traversed, as well as road accessibility characteristics (Supplementary Table 1). A baseline walking speed was set a 4 km/hour, in keeping with other geospatial access studies. The model adjusts walking speeds for elevation based upon Tobler’s hiking function, which incorporates the angle of slope of the underlying terrain. For roads classified by the UN logistics cluster as “difficult to access,” the allowed travel speeds were reduced by 50%. Speeds were verified with local staff. Finally, given the inherent uncertainty around the speed parameters and the dynamic nature of travel conditions in conflict settings, we performed sensitivity analyses for each model, varying the assigned speed for each landcover or road type by 20% to create upper and lower sensitivity bounds, consistent with other studies^23^.

Conflict and context-specific considerations were explicitly incorporated in the model as follows: (1) As described, the road network was modified to reflect conflict-altered road conditions as determined by the UN Yemen Logistics Cluster, the most reliable, timely source of transportation data for UN partners; (2) Updated population data were developed by WorldPop using revised UN OCHA population estimates, which incorporate data on internal displacement; and (3) *mahram* requirements that restrict women and girls from travelling outside of their home governorate in many areas were incorporated by treating governorate boundaries as fixed barriers.

The accessibility analysis generated a spatial raster showing the geographic extent of areas covered within the defined travel time thresholds, and tabular data on the number and percentage of people living within the defined travel times, disaggregated by specified geographic boundaries (e.g., governorates). For each analysis, we calculated the number and proportion of the under-5 population living within the 30-minute walk time of the relevant facilities or mobile teams at both the national and governorate level. We also calculated these values for other time thresholds to illustrate the change in accessibility by time.

To explore other dimensions of geographic accessibility, we also performed two supplementary analyses. First, we modified the original travel scenario to allow for motorized transport when travelling on roads (Supplementary Table 2). While less relevant in the Yemen context due to limited population access to motorized transport (see introduction), the motorized transport model does provide another perspective on accessibility that may be relevant in some locations. For this model, driving speeds were based on the road type being traversed, as well as whether the road segment was deemed “difficult to access.” Second, we performed a population-weighted travel-time reduction analysis, following published methodology^25^, to map areas where populations have the largest reduction in overall “person- travel time” due to mobile clinics. In this analysis, we applied a 1 km^2^ grid to the travel time and population distribution rasters generated from both walking scenarios (“fixed site only” and “fixed site and mobile clinic”), then used zonal statistics to derive the mean travel time (in minutes) and population sum for each 1 km^2^ grid-cell. These values were multiplied to obtain the mean person travel time (in minutes) per grid-cell. The reduction in mean person travel time per grid-cell due to mobile clinics was obtained by subtracting the values from the “fixed site and mobile clinic” scenario from the “fixed site only” scenario.

Finally, we performed a series of scale-up analyses to select new mobile sites, with the explicit goal of optimizing the number of children within a 30-minute walk of the nearest vaccination site. Based on meetings with local partners and political realities, the following context-informed criteria were adopted: (1) New sites must be greater than a 30 min walk time from an existing facility or mobile health team site already offering EPI; (2) Priority should be given to locations with the highest population density within a small (<5 km) radius of the proposed site, excluding populations already covered by another site; (3) No more than one new site is allowed per sub-district, to limit geographic clustering and promote geographic equity. Stakeholders requested that the analysis generate the top 300 options, reflecting current funding envelopes and unit costs for mobile teams. To perform the scale-up analysis, we first generated a residual population raster from AccessMod, representing the spatial distribution of the under-5 population located beyond a 30-minute walk of the nearest existing fixed or mobile vaccination site. Next, in QGIS, we overlaid a 1 km^2^ grid, clipped to match the geographic extent of the residual population. For each grid-cell, zonal statistics were performed to derive the residual population and average travel time to the nearest EPI site. Spatial joins were performed to assign sub-district codes and geospatial coordinates to each grid-cell. The grid-cell list was filtered to retain one grid-cell per sub-district with the highest residual population. From this list, the 300 grid-cells with the largest residual population were selected (Supplementary Data File 1). Their geo-coordinates were added to the list of existing fixed and mobile vaccination sites, and a revised accessibility model was run in AccessMod estimating the impact of adding these scale-up sites on the number of children living within a 30-minute walk of vaccination. Additionally we compared the results of using 1 km^2^, 3 km^2^ and 5 km^2^ grids in the selection process to determine which version generated site locations yielding the highest gains in population access (Supplementary Table 5).

To map areas with the greatest reduction in person-travel time due to the new scale-up sites, we performed a population-weighted version of this analysis, again following a previously published methodology^25^. Mean person-travel times (in person-minutes) per 1 km^2^ grid cell were calculated for the baseline (“fixed and mobile site”) and scale-up scenarios by multiplying the mean travel time and population in each grid-cell. The difference in values between the two scenarios represents the reduction in person-minutes due to the scale-up sites. Lastly, to assess whether the distribution of current mobile sites could be further optimized, we calculated the number and percent of existing mobile sites that fall within a 30-minute walk time catchment of a health facility offering EPI services.

### Reporting summary

Further information on research design is available in the Nature Portfolio Reporting Summary linked to this article.

## Results

**Fig. 1:**
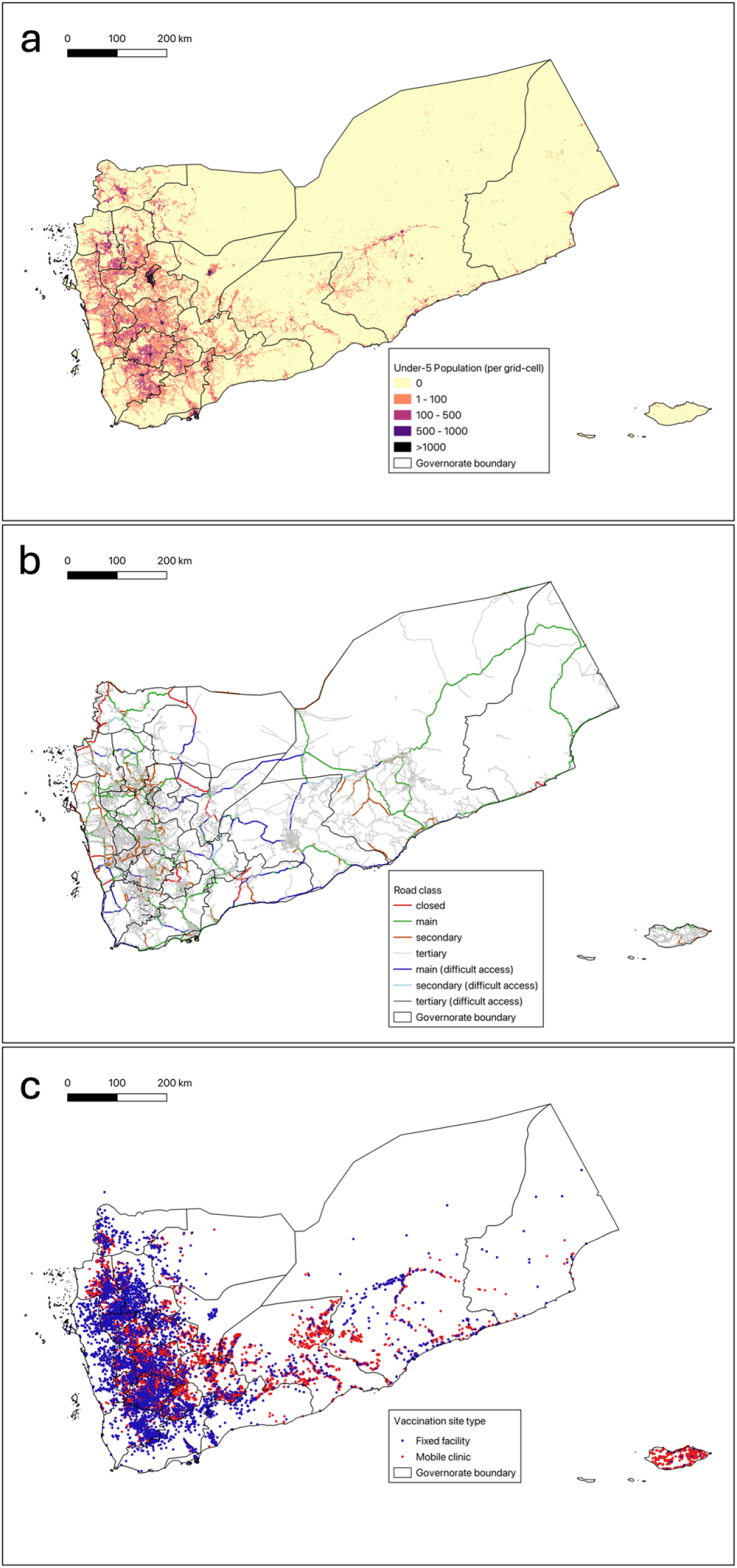
Key geospatial data inputs for modelling childhood vaccination access in Yemen. **a** Spatial population distribution raster for children aged 0–4 in Yemen, based on 2023 UN OCHA estimates and updated WorldPop population modelling. Values resampled at 1 km^2^ resolution for visualization. **b** Road network in Yemen, 2023, based on Open Street Map base network and updated using UN Logistics Cluster data to account for closed and difficult to access roads. **c** Map of fixed facilities and mobile sites offering EPI services in Yemen, 2023. Blue circles represent fixed health facilities offering vaccinations, while red circles represent locations of mobile health clinics offering vaccinations.

We identified 3750 fixed facilities with verified geocoordinates offering EPI services in Yemen 2023, according to the HeRAMS dataset. Of these, 2445 were primary healthcare units, 1073 were primary healthcare centres, and 233 were hospitals. We identified 6,365 additional EPI sites with verified geocoordinates served by UNICEF-supported mobile health teams. These sites, along with the custom population distribution raster and road network grid, are shown in Fig. 1a–c.

### Geographic access to EPI services

**Fig. 2:**
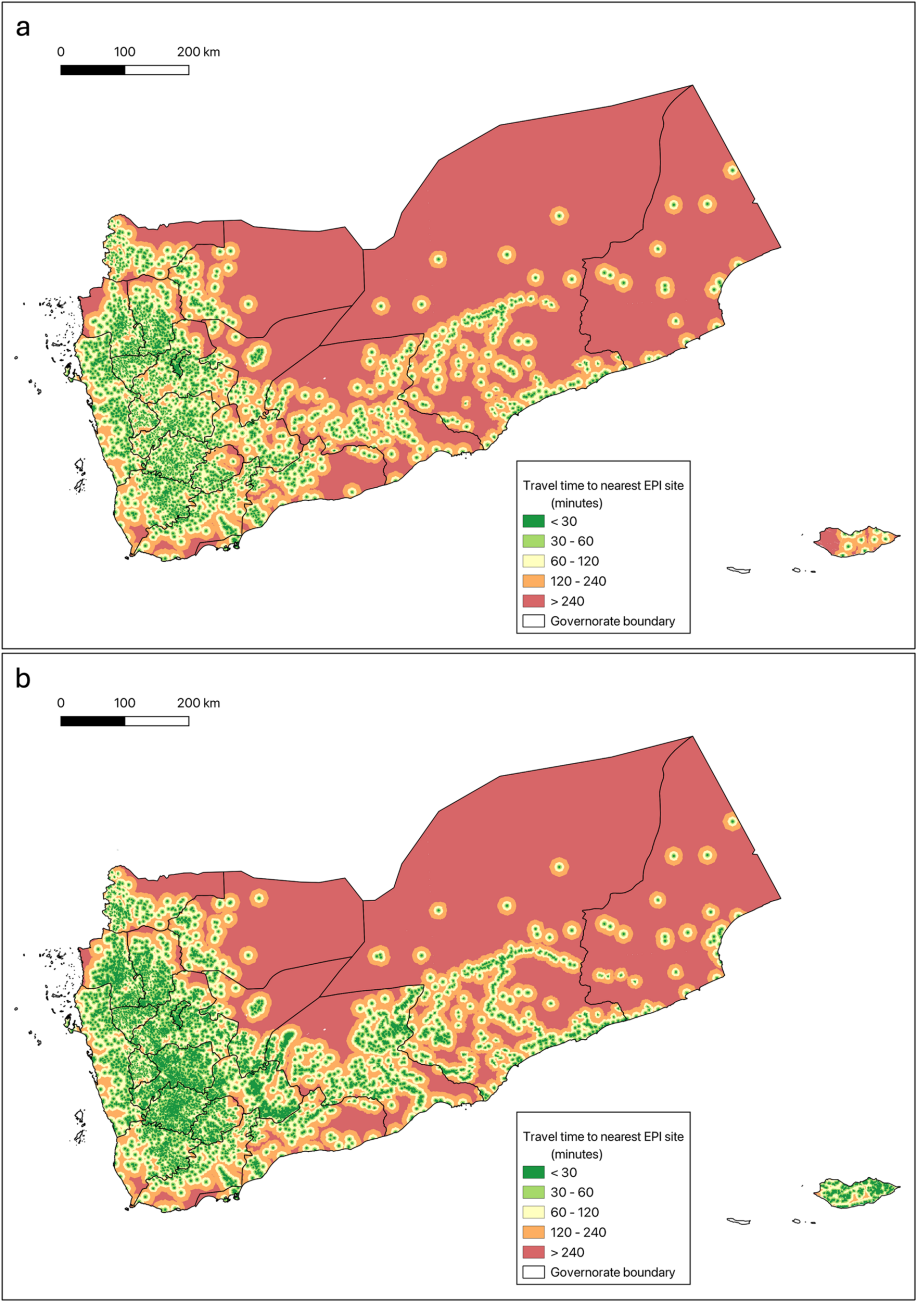
Estimates of travel times to vaccination sites in Yemen. **a** Map of travel times to the nearest fixed health facility offering vaccinations**. b** Map of travel times to the nearest fixed health facility or mobile health team site offering vaccinations.

Figure. 2 shows the geographic extent of areas within defined walk times, ranging from less than 30 min to greater than four hours, of the nearest fixed facility offering EPI services (a), as well as the geographic extent of areas within the same walk-time thresholds to the nearest fixed facility or mobile health team site (b).

At the national level, we find that 3.05 million children (56.5% of the Yemen under-5 population) live within a 30-minute walk of a fixed facility offering EPI services. By comparison, we find that 3.57 million children (66.3% of the under-5 population) live within a 30-minute walk of either a fixed facility or mobile clinic, indicating that mobile service delivery currently augments EPI access by roughly 525,000 children, or 9.8% of the under-5 population.

At the governorate level, we find substantial variability in access. Table 1 shows the number of children under age 5 and the percentage of the under-5 population living within a 30-minute travel time of EPI services, disaggregated by governorate. For fixed facility services only, we find that the percentage of the under-5 population within a 30-minute walk of EPI service ranged from a high of 87.9% and 72.5% in Sana’a City and Aden, to a low of 4.8% and 37.2% in Socotra and Raymah governorates, respectively. In 10 of 22 governorates, less than 50% of the population lived within a 30-minute walk of a facility offering EPI.

**Table 1:** Number and percent of children under 5 living within a 30-minute walk of EPI services, by delivery modality and governorate

Governorate	Total population	Fixed sites only				Fixed and Mobile Sites				Difference	
		No. children within 30 min walk of EPI site	Uncertainty intervals (+/− 20% travel speed)	Percent of under-5 governorate population	Uncertainty intervals (+/− 20% travel speed)	No. children within 30 min walk of EPI site	Uncertainty intervals (+/− 20% travel speed)	Percent of under-5 governorate population	Uncertainty intervals (+/− 20% travel speed)	Difference (%) between scenarios	Uncertainty intervals (+/− 20% travel speed)
Ibb	448,293	216,034	(172,673, 254,482)	48.2	(38.5, 56.8)	318,946	(265,637, 355,580)	71.1 (59.3, 79.3)	(59.3, 79.3)	23	(20.8, 22.5)
Abyan	72,069	42,705	(35,835, 47,737)	59.3	(49.7, 66.2)	44,681	(37,797, 49,469)	62.0 (52.4, 68.6)	(52.4, 68.6)	2.7	(2.7, 2.4)
Sana’a City	835,434	734,375	(675,511, 774,340)	87.9	(80.9, 92.7)	739,917	(681,439, 779,799)	88.6 (81.6, 93.3)	(81.6, 93.3)	0.7	(0.7, 0.6)
Al Bayda	121,009	61,327	(51,154, 69,610)	50.7	(42.3, 57.5)	98,088	(89,564, 103,433)	81.1 (74.0, 85.5)	(74.0, 85.5)	30.4	(31.7, 28)
Ta’iz	483,149	243,367	(195,894, 287,586)	50.4	(40.5, 59.5)	266,489	(215,409, 313,022)	55.2 (44.6, 64.8)	(44.6, 64.8)	4.8	(4.1, 5.3)
Al Jawf	120,037	61,945	(51,687, 71,213)	51.6	(43.1, 59.3)	65,984	(55,449, 74,931)	55.0 (46.2, 62.4)	(46.2, 62.4)	3.4	(3.1, 3.1)
Hajjah	419,576	225,757	(185,423, 256,558)	53.8	(44.2, 61.1)	290,614	(246,872, 324,202)	69.3 (58.8, 77.3)	(58.8, 77.3)	15.5	(14.6, 16.2)
Al Hodeidah	607,659	312,567	(268,987, 351,710)	51.4	(44.3, 57.9)	318,711	(274,586, 358,691)	52.4 (45.2, 59.0)	(45.2, 59.0)	1	(0.9, 1.1)
Hadramawt	240,086	153,527	(138,448, 164,723)	63.9	(57.7, 68.6)	164,234	(148,811, 174,750)	68.4 (62.0, 72.8)	(62.0, 72.8)	4.5	(4.3, 4.2)
Dhamar	464,031	200,745	(163,653, 235,348)	43.3	(35.3, 50.7)	318,844	(275,640, 352,926)	68.7 (59.4, 76.1)	(59.4, 76.1)	25.5	(24.1, 25.4)
Shabwah	115,179	51,652	(42,882, 58,904)	44.8	(37.2, 51.1)	81,774	(73,262, 88,039)	71.0 (63.6, 76.4)	(63.6, 76.4)	26.2	(26.4, 25.3)
Sa’dah	178,765	77,786	(60,695, 93,417)	43.5	(34.0, 52.3)	84,401	(67,084, 99,168)	47.2 (37.5, 55.5)	(37.5, 55.5)	3.7	(3.5, 3.2)
Sana’a	178,490	73,202	(56,964, 89,040)	41	(31.9, 49.9)	91,709	(73,462, 109,212)	51.4 (41.2, 61.2)	(41.2, 61.2)	10.4	(9.3, 11.3)
Aden	179,472	130,189	(115,705, 139,648)	72.5	(64.5, 77.8)	130,569	(116,542, 139,833)	72.8 (64.9, 77.9)	(64.9, 77.9)	0.2	(0.4, 0.1)
Lahj	149,306	73,680	(59,087, 86,128)	49.3	(39.6, 57.7)	75,311	(60,685, 87,610)	50.4 (40.6, 58.7)	(40.6, 58.7)	1.1	(1, 1)
Ma’rib	129,950	65,328	(51,740, 79,816)	50.3	(39.8, 61.4)	71,035	(57,259, 85,569)	54.7 (44.1, 65.8)	(44.1, 65.8)	4.4	(4.3, 4.4)
Al Mahwit	116,862	66,575	(54,085, 77,519)	57	(46.3, 66.3)	75,791	(62,089, 86,688)	64.9 (53.1, 74.2)	(53.1, 74.2)	7.9	(6.8, 7.9)
Al Maharah	42,452	18,020	(14,982, 20,529)	42.4	(35.3, 48.4)	21,947	(18,919, 24,421)	51.7 (44.6, 57.5)	(44.6, 57.5)	9.3	(9.3, 9.1)
Amran	224,843	139,965	(118,601, 156,462)	62.2	(52.7, 69.6)	158,063	(138,044, 172,724)	70.3 (61.4, 76.8)	(61.4, 76.8)	8	(8.7, 7.2)
Ad Dali’	157,272	64,649	(52,575, 75,120)	41.1	(33.4, 47.8)	101,665	(85,706, 114,561)	64.6 (54.5, 72.8)	(54.5, 72.8)	23.5	(21.1, 25)
Raymah	97,874	36,444	(28,760, 44,171)	37.2	(29.4, 45.1)	46,240	(36,598, 55,145)	47.2 (37.4, 56.3)	(37.4, 56.3)	10	(8, 11.2)
Socotra	13,339	636	(564, 1,102)	4.8	(4.2, 8.3)	9,900	(8,425, 10,478)	74.2 (63.2, 78.6)	(63.2, 78.6)	69.5	(59, 70.3)
Total	5,395,148	3,050,475	(2,595,905, 3,435,161)	56.5	(48.1, 63.7)	3,574,915	(3,089,277, 3,960,252)	66.3 (57.3, 73.4)	(57.3, 73.4)	9.8	(9.2, 9.7)

The presence of mobile health teams augmented access in many, although not all, governorates. The largest increases in access (by absolute percentage of the governorate under-5 population) were seen in Socotra (+69.5%), Al Bayda (+30.4%), Shabwah (+26.2%) and Dhamar (+25.5%). The smallest percent increases were seen in Aden (+0.2%), Sana’a City (+0.7%), Al Hodeidah (+1.0%), and Lahj (+1.1%).

Even with the contributions from mobile health teams, geographic access to vaccination sites remains poor in several governorates. We find that in Raymah and Sa’dah governorates, only 47.2% of the under-5 population lives within a 30-minute walk of a fixed facility or mobile health team offering EPI services. In 16 of 22 governorates, the percentage of the under-5 population with EPI access remains below 70%, even after accounting for the contribution of mobile services.

**Fig. 3:**
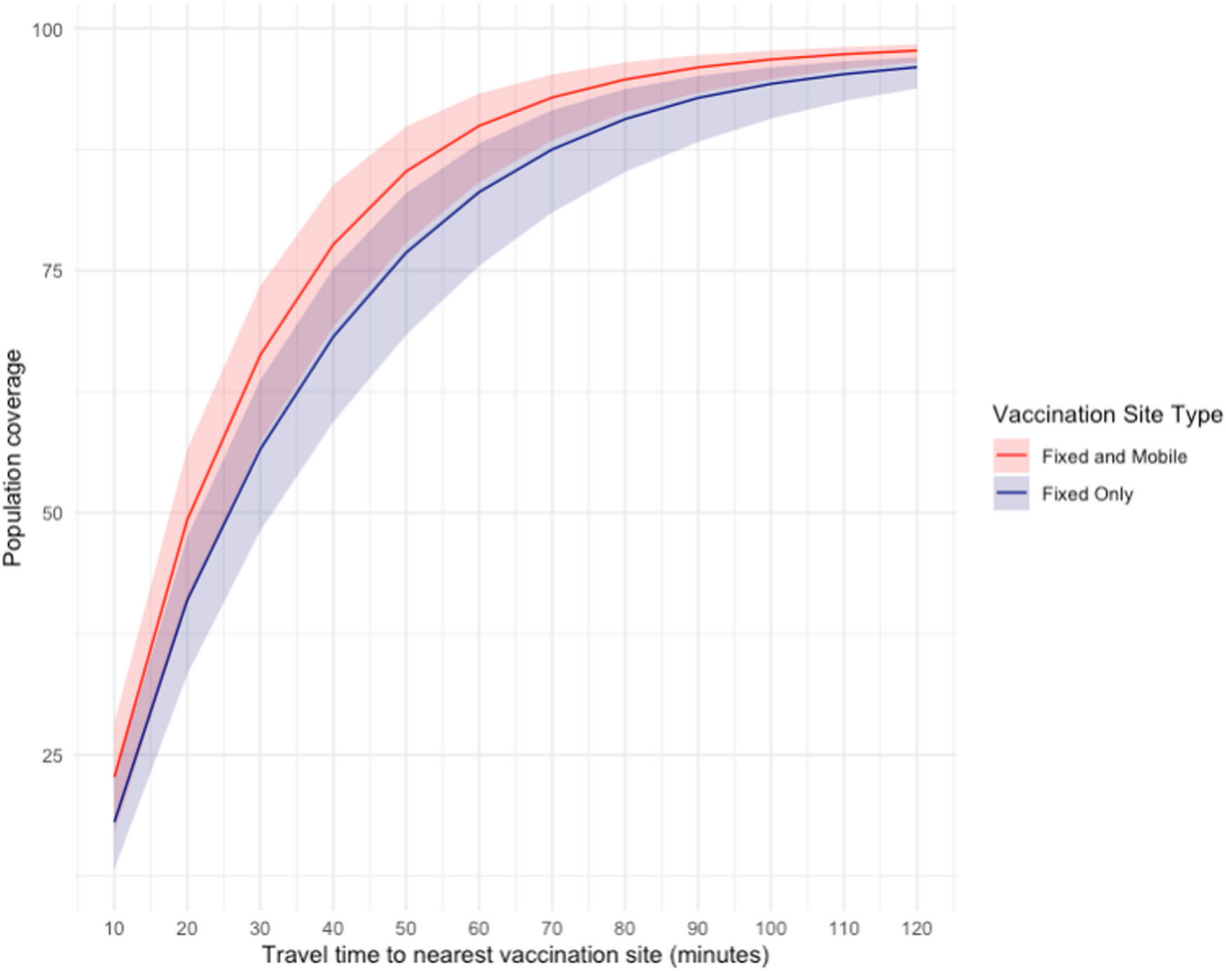
Population coverage across accessibility time thresholds. Solid lines represent the percentage of under-5 population that can reach the nearest vaccination site under different time thresholds, ranging from 10 to 120 min. Shaded regions represent uncertainty intervals, calculated using 20% slower and faster speeds from those used in the base scenario.

In addition to the 30-minute threshold, we modelled differences in accessibility based on a continuum of walking time thresholds, ranging from 10 min to 120 min, as shown in Fig. 3. At 60 min, we estimate that roughly 82% of the under-5 population can access vaccinations through fixed facilities, and more than 90% can access vaccinations at fixed or mobile facilities. At 120 min, roughly 98% of the population can access vaccinations through both modalities. We also modelled the relative contribution of mobile sites to accessibility under a travel scenario allowing motorized transportation (Supplementary Table 3).

The results of the population-weighted travel-time reduction analysis, mapping areas with the largest reduction in overall “person-travel time” due to mobile clinics, are provided in Supplementary Fig. 2.

### Scale-up analysis

**Fig. 4:**
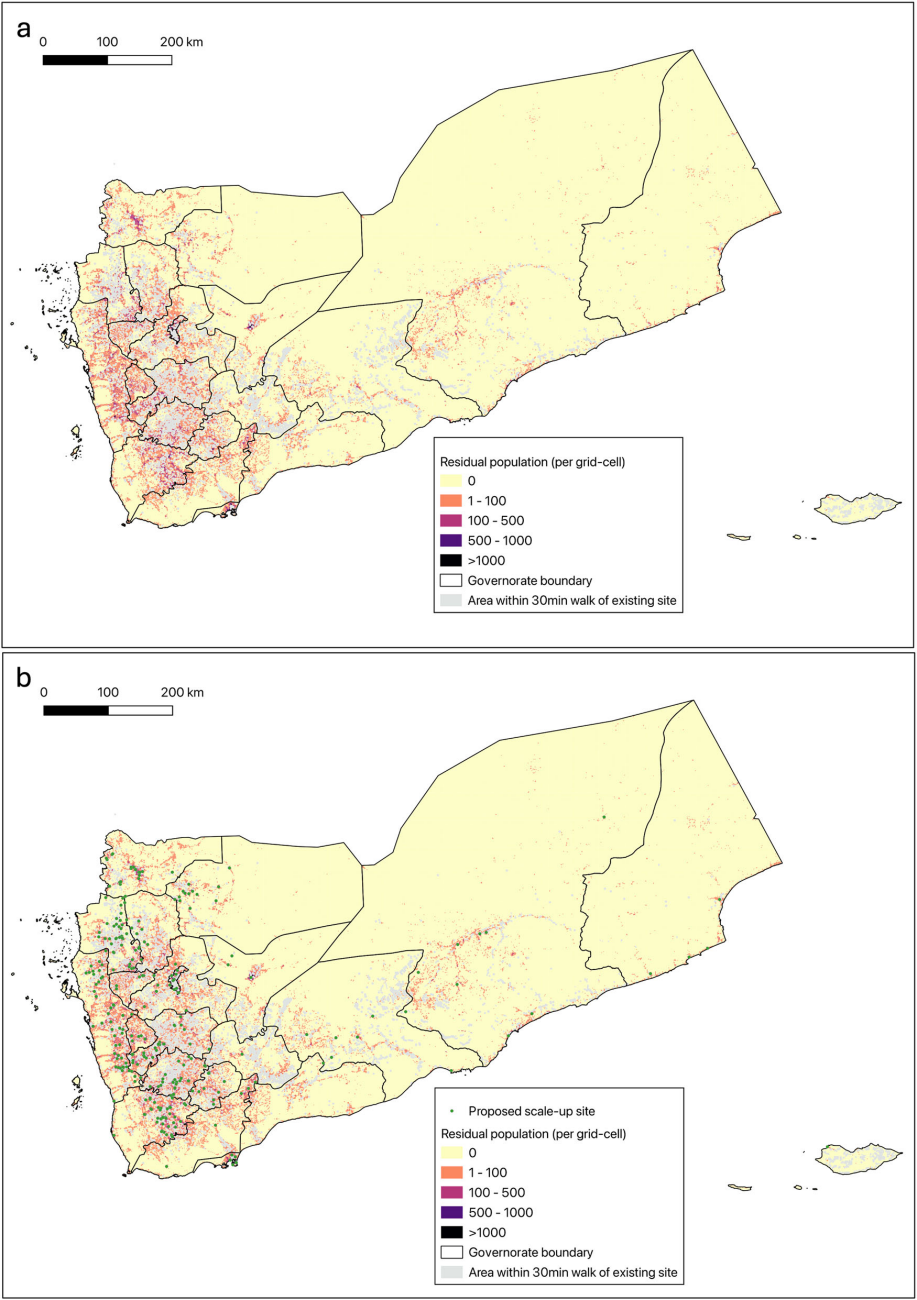
Scale-up target population and proposed scale-up sites. **a** Map of scale-up target population (residual population not within a 30-minute walk time of existing EPI site) per 1 km^2^ grid cell. Gray areas represent locations already within a 30-minute walk time of an EPI site that is masked from the scale-up analysis. **b** Proposed scale-up sites, superimposed on the residual population.

Figure. 4 shows the locations of the 300 proposed scale-up sites superimposed upon the residual population (the target population of the scale-up effort, i.e. children living beyond a 30-minute walk time of existing EPI services). These represent the 300 top-ranked sites according to the selection criteria outlined in the methodology and are based on a 1 km^2^ grid to assess new candidate sites. A full list of the selected sites, ranked by the target population within the corresponding grid-cell, is provided in Supplementary Data File 1. Supplementary Fig. 4 provides a high-resolution example of the selection process, with proposed scale-up sites overlaid on the residual target population.

Table 2 shows the impact of the addition of these new sites on the number of children within 30-minute walk times to EPI services, derived from our revised geographic accessibility model that includes the new sites as well as current fixed and mobile sites. The results are compared against the “baseline” accessibility analysis that includes only the existing fixed and mobile vaccination sites. We estimate that by adding the 300 scale-up sites, geographic access to EPI services within 30-minutes would increase by 388, 475 children, or an additional 7.2% (from 66.3% to 73.5%) of the under-5 population. This equates to an average access gain of 1,294 children per site (388,475 children/300 sites), compared to 82 children per site (524,440 children/6365 mobile sites) under the current set and distribution of mobile sites.

**Table 2:** Comparison of geographic access (population within 30-minute walk times) to vaccinations under current versus proposed scale-up scenario

Governorate	Total Population	Current Scenario		Scale-up Scenario		Difference	
		No. children within 30 min walk of EPI services	Percent of Governorate Under-5 population	No. children within 30 min walk of EPI services	Percent of Governorate Under-5 population	No. children	Percent
Ibb	448,293	318,946	71.1	343,318	76.6	24,372	5.5
Abyan	72,069	44681	62.0	45065	62.5	384	0.5
Sana’a City	835,434	739,917	88.6	787,484	94.3	47,567	5.7
Al Bayda	121,009	98,088	81.1	99,239	82.0	1151	0.9
Ta’iz	483,149	266,489	55.2	315,000	65.2	48,511	10.0
Al Jawf	120,037	65,984	55.0	80,757	67.3	14,773	12.3
Hajjah	419,576	290,614	69.3	327,263	78.0	36,649	8.7
Al Hodeidah	607,659	318,711	52.4	379,423	62.4	60,712	10.0
Hadramawt	240,086	164,234	68.4	176,008	73.3	11,774	4.9
Dhamar	464,031	318,844	68.7	344,677	74.3	25,833	5.6
Shabwah	115,179	81,774	71.0	87,580	76.0	5806	5.0
Sa’dah	178,765	84,401	47.2	110,154	61.6	25,753	14.4
Sana’a	178,490	91,709	51.4	99,667	55.8	7958	4.4
Aden	179,472	130,569	72.8	150,727	84.0	20,158	11.2
Lahj	149,306	75,311	50.4	81,515	54.6	6204	4.2
Ma’rib	129,950	71,035	54.7	82,652	63.6	11,617	8.9
Al Mahwit	116,862	75,791	64.9	79,469	68.0	3678	3.1
Al Maharah	42,452	21,947	51.7	28,202	66.4	6255	14.7
Amran	224,843	158,063	70.3	168,885	75.1	10,822	4.8
Ad Dali’	157,272	101,665	64.6	115,063	73.2	13,398	8.6
Raymah	97,874	46,240	47.2	49,234	50.3	2994	3.1
Socotra	13,339	9900	74.2	12,006	90.0	2106	15.8
Total	5,395,148	3,574,915	66.3	3,963,390	73.5	388,475	7.2

Using this approach, the largest governorate-level increases in EPI access, by percentage, are seen in Socotra (15.8% gain), Sa’dah (14.4%), Al Jawf (12.3%), and Aden (11%). By absolute gain, the largest increases were seen in Ta’iz (48,511), Sana’a City (47,567), and Ibb (24,372) governorates.

A map of reduction in person-travel time was also calculated for each 1 km grid-cell to help illustrate locations with sizable populations where travel times decreased substantially as result of the new sites (Supplementary Fig. 3). Additionally, Supplementary Table 5 shows how using a 1 km^2^ grid in the modelling process to select sites results in greater accessibility gains that a 3 km^2^ or 5 km^2^ grid.

Finally, to further inform future vaccination delivery planning, we assessed the extent to which mobile sites fall within the 30-minute walk catchment of facility-based EPI services. We find that 1781 of the 6365 current mobile sites (28%) are located within a 30-minute walk time of a fixed facility already offering EPI services (Supplementary Table 4). More than half of these mobile sites are in just three governorates: Dhamar (424 sites), Shabwah (307 sites), Al Bayda (203 sites).

## Discussion

This analysis finds that mobile service delivery substantially augments the number and proportion of children in Yemen with geographic access to vaccinations, compared to fixed facilities alone, although the impact is not distributed evenly across governorates. Some governorates see much greater gains in EPI access, based upon current mobile clinic programming levels and distribution. We find that the addition of carefully selected scale-up sites would result in large increases in geographic access, but even with these gains, notable access gaps persist. Specifically, we find that adding 300 new sites based upon carefully defined, contextually relevant criteria still leaves more than a quarter of the under-5 population beyond a 30 min walk time of a vaccination site.

These findings have direct implications for current programming decisions and national dialogues on vaccination coverage in Yemen. Despite concerted efforts by international stakeholders and health officials, vaccination rates remain concerningly low, and recent outbreaks – including a polio outbreak that claimed more than 270 lives over the past three years – underscore the consequences of declining coverage. With large-scale vaccination campaigns restricted in the current geopolitical context, stakeholders have identified mobile clinics as a critical component of vaccination delivery in the country. By quantifying the contribution of mobile teams to current geographic vaccine access, we identified with a high degree of spatial resolution which areas remain underserved by any type of vaccination programme – fixed or mobile. Moreover, our modelling allows us to identify and map where expanding mobile clinics might benefit the greatest number of children who currently live greater than a 30-minute walk to the nearest vaccination site, and to quantify the impact of scaling-up at EPI services at these sites. Additionally, it highlights the importance of re-examining the geographic distribution of current programming, given that more than a quarter of current mobile sites fall within the 30-minute walk catchments of existing facilities offering EPI. This pattern suggests that some mobile sites might not be optimally distributed and aligned with national strategy. This information is already being used by country health authorities and UN partners to inform vaccination planning efforts for the coming year.

More broadly, these findings highlight critical questions facing agencies and donors over the design, implementation, and impact of mobile clinics in humanitarian settings. The literature on mobile clinics is largely limited to case studies, even as stakeholders demonstrate a growing appetite to deploy them^4–8^. These case studies nonetheless raise important considerations around community acceptance, monitoring and evaluation, service quality, and sustainability. One study from the DRC, for example, highlighted the importance of recurring visits for engendering community trust and mobile service uptake^16^. Other studies emphasized the importance of routinely engaging local partners to improve community acceptance^10^. A case study in Haiti commented on the challenge of ensuring high-quality services through mobile approaches, particularly if specialized equipment or technology is required^13^. In Yemen, community involvement and recurring visits have long been part of the design of mobile programming, with local leaders participating in site selection, planning, and community engagement. As Yemeni health officials and stakeholders work to strengthen vaccination coverage, these considerations should be central to any future scale-up activities. Moreover, given the scale of mobile activities in Yemen, it is critical that these modalities continue be studied, not only to improve implementation locally but also to contribute to pool of knowledge around mobile service provision in humanitarian contexts globally.

As the issue of community acceptance attests, geographic access, while the focus of this analysis, is far from the only determinant of vaccination uptake and coverage. Individuals who live within reasonable distances of sites that offer care may not seek it out due to concerns about affordability, quality, cultural appropriateness, and lost revenue from missing work^7,8^. Other demand factors may also play a significant role in limiting vaccination-seeking behaviour. For example, parents may hold inaccurate beliefs about vaccine safety or efficacy. Given this reality, an important next step would be to examine whether modelled geographic access aligns with realized vaccination coverage. Areas with high geographic access but low real-world coverage might warrant further investigation of the factors contributing to low uptake. Similarly, analyzing whether coverage rates in areas served primarily by mobile services are similar to those served by fixed facilities would help measure the realized effectiveness of mobile clinics, i.e. whether mobile clinics are truly addressing the delivery gaps they are intended to fill. Qualitative studies exploring household attitudes on vaccination might be particularly helpful in this regard.

Another important future direction would involve evaluating cost-effectiveness. Studies from other conflict settings have shown that mobile services can be more expensive than those offered at fixed facilities and difficult to sustain financially, particularly when donor funding decreases^12^. Such studies are critical for programme planners, but often not done^11^. Ad hoc mobile clinics can also pose challenges to broader health system strengthening efforts and decision-making around funding for service delivery. Future work could involve a costing exercise, including exploring the relative cost-benefit of fixed versus mobile service delivery, as well as modelling the cost and impact of different scale-up scenarios (e.g., expanding service availability at fixed facilities not currently offering EPI versus continuing mobile services in those areas). These calculations would need to account not only for vaccinations, but also the other health services provided by these teams. Indeed, with the protracted nature of the Yemen conflict and increasing pressure on all stakeholders to support sustainable interventions, stakeholders are now exploring the idea of creating quasi-permanent health posts in locations currently supported by mobile health teams. The analytical work presented here could help guide such decisions and provide a methodologically rigorous, evidence-based approach to future service planning.

This analysis has additional limitations. Conflict landscapes are dynamic, and although we account for population internal displacement, road network status, and other context-relevant developments (such as movement restrictions across governorates) by using updated and original datasets, these inputs may not reflect the most recent local developments. Future iterations of this work could involve additional ground-truthing activities at the local level, including consulting local health authorities about changes in facility status or road network navigability. Second, we use 30-minute walk times as our threshold for access to EPI services. This decision was based upon internal data showing that most beneficiaries walk to care (i.e. do not use or do not have access to motorized transport) and travel less than 30 min to primary care services, as well as long-standing government strategy that focuses on using outreach or mobile programmes to reach communities beyond 30 min of a health facility. By using this arguably conservative definition, we are potentially underestimating access for populations that use motorized transport or are willing travel further distances to access primary care services. Future household surveys or qualitative analyses could incorporate questions to better assess caregivers’ willingness to travel to obtain vaccinations for their children.

Finally, the use of geospatial tools to identify locations for scale-up is one that warrants careful reflection, particularly with regards to the choice of metric for optimization. In this analysis, we focus on optimizing the number of children within a 30-minute walk time of a vaccination facility. This decision was based on survey results and extensive dialogue with in-country partners reflecting the care-seeking behaviours of Yemeni beneficiaries. Alternatively, one could seek to optimize reductions in person-travel times, i.e. reducing the travel time to the nearest site by the greatest amount for the greatest number of people. There are trade-offs with either choice: the former may benefit administrative regions with denser populations over those with more dispersed populations, while the latter may decrease travel times for a large number of people but result in relatively fewer people meeting certain time travel cut-offs (e.g., 30 min). Ultimately, the question of what parameter to optimize is a context-specific one, based upon local needs, health system priorities, and care-seeking behaviours. Regardless, as this analysis and others demonstrate, geospatial tools provide a powerful approach for understanding these dynamics and informing health system planning, even in complex humanitarian contexts.

In conclusion, this study, to our knowledge, is the first study to quantify the national and sub-national impact of mobile services on vaccination access in a humanitarian or protracted conflict setting. We use conflict-adjusted data, contextually informed scenarios, and geospatial modelling to identify locations to optimize future mobile team scale-up activities. We find that mobile programming increases geographic access to childhood vaccination services in Yemen and plays a critical role in reaching populations that would otherwise have limited access to routine vaccination, particularly in the current geopolitical context. Moreover, we demonstrate that scaling up mobile services in a modest number of appropriately selected sites would lead to substantial further improvements in geographic access, while still leaving large gaps. These findings can directly inform national dialogues and decision-making to improve vaccination and basic primary care coverage.

## Supplementary information

Supplementary Information

Description of Additional Supplementary Files

Supplementary Data 1

Supplementary Data 2

Reporting Summary

## Data Availability

Health facility and mobile site datasets are not publicly available due to the sensitive nature of the geospatial coordinates; access requests can be made to the corresponding author. The base road network dataset can be downloaded from https://data.humdata.org/dataset/hotosm_yem_roads^29^. Elevation data can be obtained from the Shuttle Radar Topography Mission (SRTM) at 10.5069/G9445JDF^30^. Land cover data can be accessed from ArcGIS Living Atlas at https://livingatlas.arcgis.com/en/home^31^. Yemen UN logistics cluster data on road closure can be obtained from https://www.logcluster.org/en/ops/yem10a^32^. Finally, the administrative boundaries are available for download from the Humanitarian Data Exchange at https://data.humdata.org/dataset/yemen-admin-boundaries^33^. The customized population and road network datasets can be shared upon request. All other data are available from the author on reasonable request. The numerical results (source data) underlying Fig. 3 are in Supplementary Data 2.
