# Supplementary material for: Geospatial analysis and scale-up modelling of the impact of mobile programming on access to essential childhood vaccinations in Yemen
Source: Commun Med (Lond). 2025 Apr 18;5:126. doi: 10.1038/s43856-025-00762-5 (PMC12008410; doi:10.1038/s43856-025-00762-5)
Supplement: Supplementary file 1 — Supplementary Information [file 43856_2025_762_MOESM1_ESM.pdf]

SUPPLEMENTARY INFORMATION

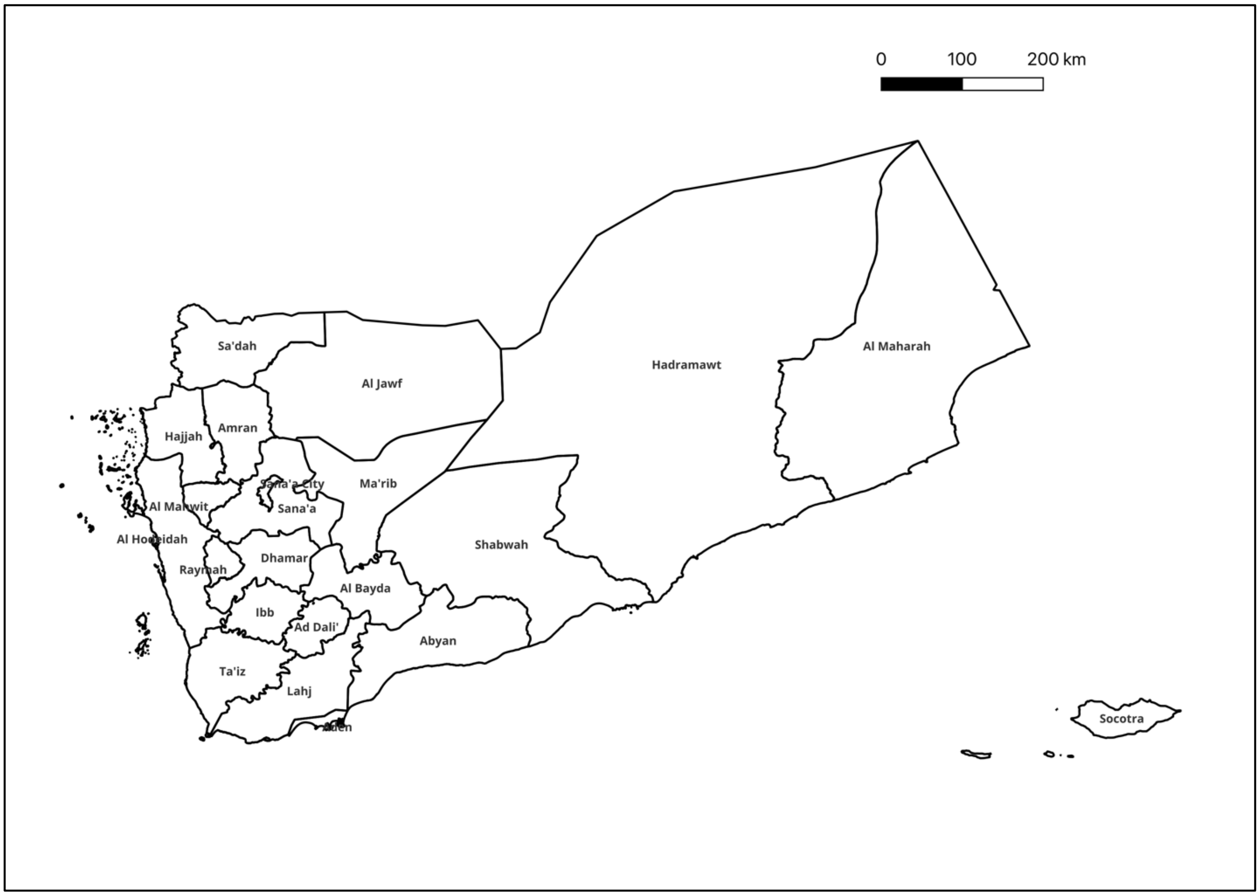

Supplementary Figure 1. Map of Yemen’s 22 governorates.

Travel Scenario – Walking only					
**class**	label	speed (km/h)	lower bound (km/h)	upper bound (km/h)	mode
**1**	Water	1	0.8	1.2	WALKING
**2**	Trees	2	1.6	2.4	WALKING
**4**	Flooded vegetation	2	1.6	2.4	WALKING
**5**	Crops	3	2.4	3.6	WALKING
**7**	Built Area	2.5	2	3	WALKING
**8**	Bare ground	4	3.2	4.8	WALKING
**9**	Snow/Ice	1.5	1.2	1.8	WALKING
**10**	Clouds	4	3.2	4.8	WALKING
**11**	Rangeland	4	3.2	4.8	WALKING
**1001**	Main road	4	3.2	4.8	WALKING
**1002**	Secondary road	4	3.2	4.8	WALKING
**1003**	Tertiary road	4	3.2	4.8	WALKING
**1006**	Main road (difficult to access)	2	1.6	2.4	WALKING
**1007**	Secondary road (difficult to access)	2	1.6	2.4	WALKING
**1008**	Tertiary road (difficult to access)	2	1.6	2.4	WALKING

Supplementary Table 1. Travel speeds by land cover and road category used in the walking-only geospatial accessibility models, with lower and upper uncertainty adjustments representing 20% slower and faster speeds, respectively, for each land cover and road category.

Travel Scenario – Motorized transport allowed					
**class**	label	speed (km/h)	lower bound (km/h)	upper bound (km/h)	mode
**1**	Water	1	0.8	1.2	WALKING
**2**	Trees	2	1.6	2.4	WALKING
**4**	Flooded vegetation	2	1.6	2.4	WALKING
**5**	Crops	3	2.4	3.6	WALKING
**7**	Built Area	2.5	2	3	WALKING
**8**	Bare ground	4	3.2	4.8	WALKING
**9**	Snow/Ice	1.5	1.2	1.8	WALKING
**10**	Clouds	4	3.2	4.8	WALKING
**11**	Rangeland	4	3.2	4.8	WALKING
**1001**	Main road	70	56	84	MOTORIZED
**1002**	Secondary road	50	40	60	MOTORIZED
**1003**	Tertiary road	30	24	36	MOTORIZED
**1006**	Main road (difficult to access)	35	28	42	MOTORIZED
**1007**	Secondary road (difficult to access)	25	20	30	MOTORIZED
**1008**	Tertiary road (difficult to access)	15	12	18	MOTORIZED

Supplementary Table 2. Travel speeds by land cover and road category used in the motorized transport-permitted geospatial accessibility model, with lower and upper uncertainty adjustments representing 20% slower and faster speeds, respectively, for each land cover and road category.

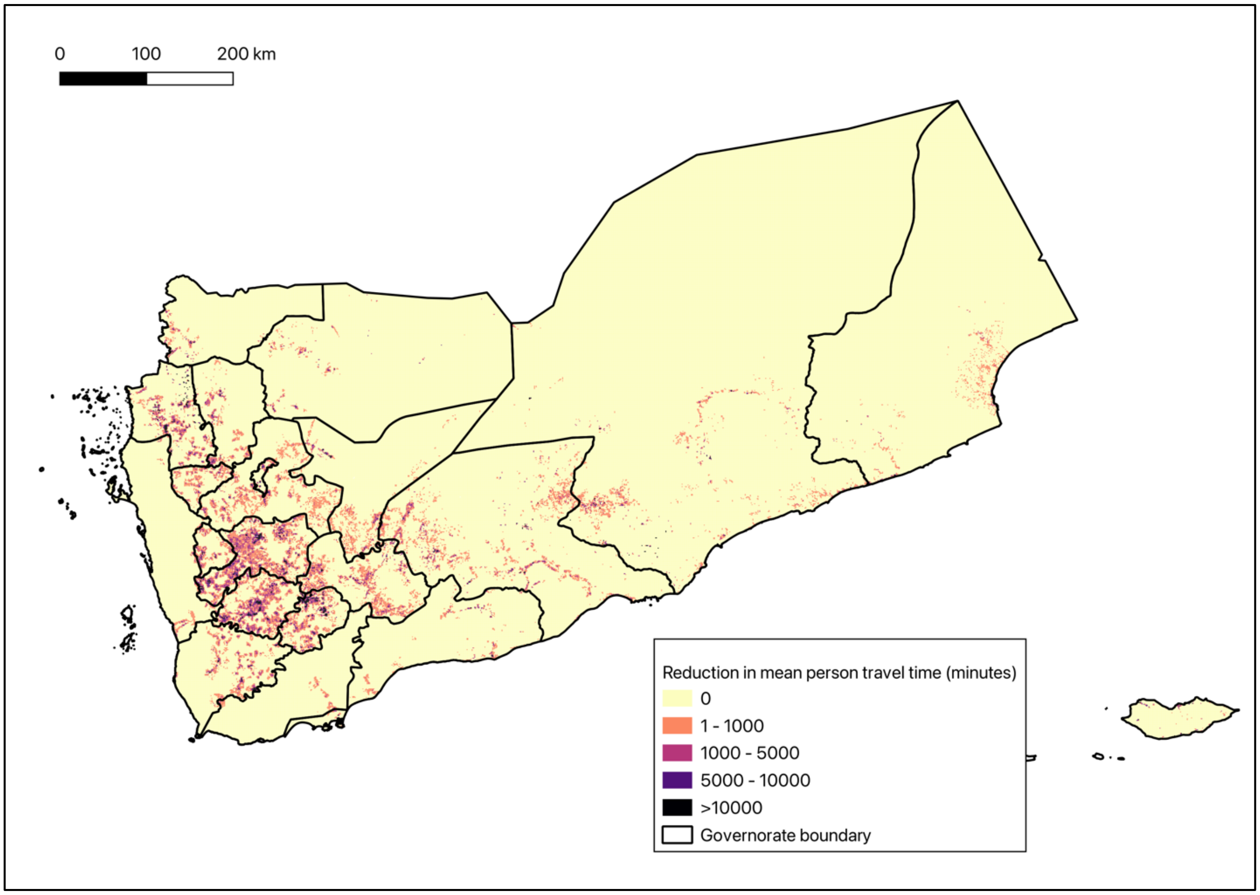

Supplementary Figure 2. Map of population-weighted travel time reduction due to mobile clinics. Values were obtained for each 1km^2 grid-cell by multiplying the population by the mean travel time to the nearest vaccination site, comparing the fixed facility only scenario to the fixed facility plus mobile site scenario. The difference in values, shown here, represents the reduction in person-travel time in each grid-cell due to mobile sites. Darker areas represented locations with the largest travel time reductions in person-time.

Under-5 population within 30-minute travel times (allowing motorized transport) of nearest vaccination site					
Governo-rate	Total Popula-tion	Fixed Sites Only		Fixed and Mobile Sites	
		No. children within 30 min travel of EPI services	% of governorate under-5 population	No. children within 30 min travel of EPI services	% of governorate under-5 population
Ibb	448293	435743	97.2	440421	98.2
Abyan	72069	64899	90.1	65151	90.4
Sana a City	835434	834343	99.9	834358	99.9
Al Bayda	121009	112150	92.7	116921	96.6
Ta iz	483149	466159	96.5	469106	97.1
Al Jawf	120037	89406	74.5	91023	75.8
Hajjah	419576	378644	90.2	392275	93.5
Al Hodeidah	607659	571794	94.1	572223	94.2
Hadramawt	240086	219423	91.4	224081	93.3
Dhamar	464031	452328	97.5	458993	98.9
Shabwah	115179	95289	82.7	104447	90.7
Sa dah	178765	159482	89.2	161577	90.4
Sana a	178490	167092	93.6	170459	95.5
Aden	179472	177767	99.0	177767	99.0
Lahj	149306	137638	92.2	137805	92.3
Ma rib	129950	120288	92.6	121568	93.5
Al Mahwit	116862	113432	97.1	114040	97.6
Al Maharah	42452	34862	82.1	35931	84.6
Amran	224843	211830	94.2	213881	95.1
Ad Dali	157272	133900	85.1	146006	92.8
Raymah	97874	93490	95.5	94424	96.5
Socotra	13339	6796	50.9	12882	96.6
**Total**	**5395148**	**5076752**	**94.1**	**5155337**	**92.8**

Supplementary Table 3: Geospatial access to vaccinates sites, comparing fixed sites only versus fixed and mobile sites, allowing access to motorized transport.

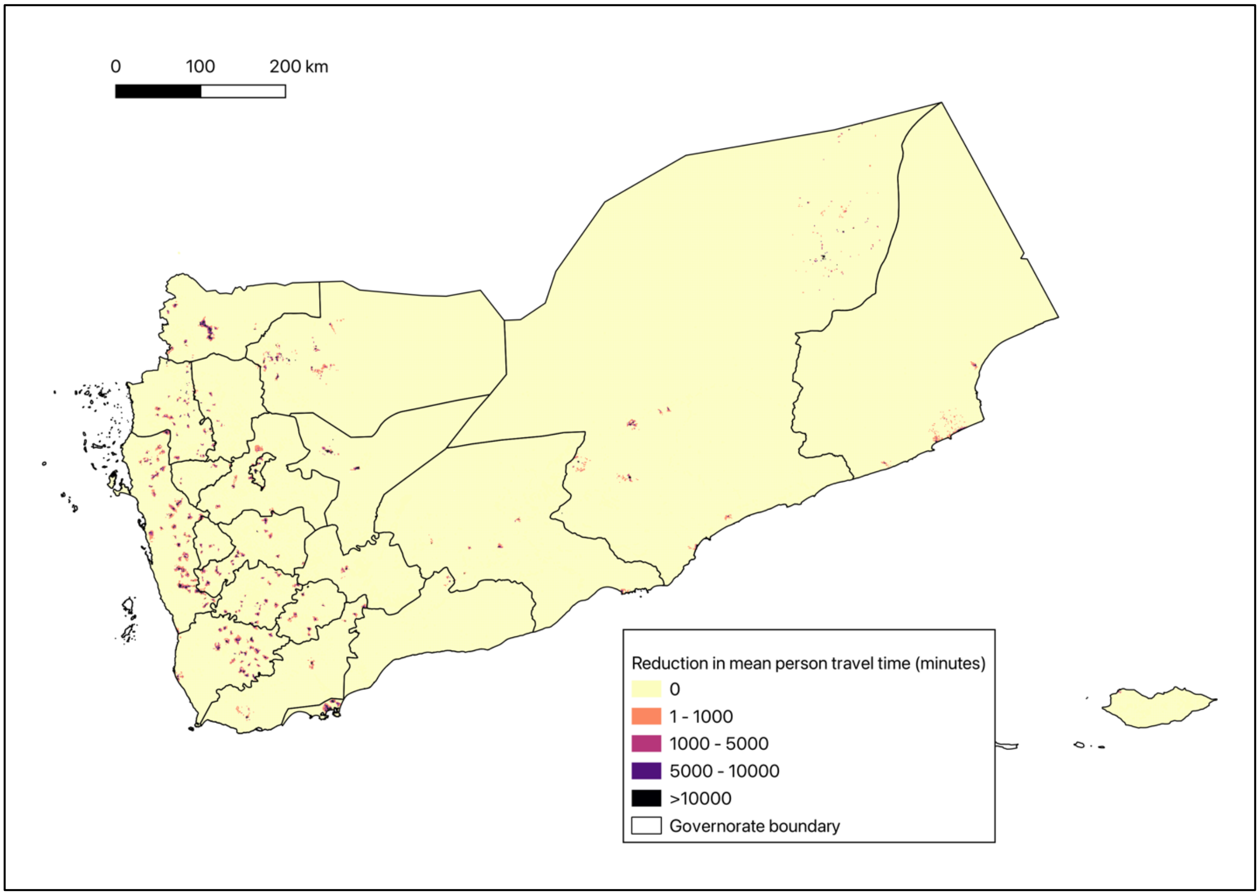

Supplementary Figure 3. Map of population-weighted travel time reduction due to 300 scale-up sites. Values were obtained by multiplying the mean travel time and population in each grid-cell, comparing the baseline ("fixed facility plus mobile site") scenario to the scale-up scenario, the latter of which includes 300 new sites. The difference in these values, shown here, represents the reduction in person-travel time in each grid-cell due to scale-up sites. Darker areas represented locations with the largest travel time reductions in person-time.

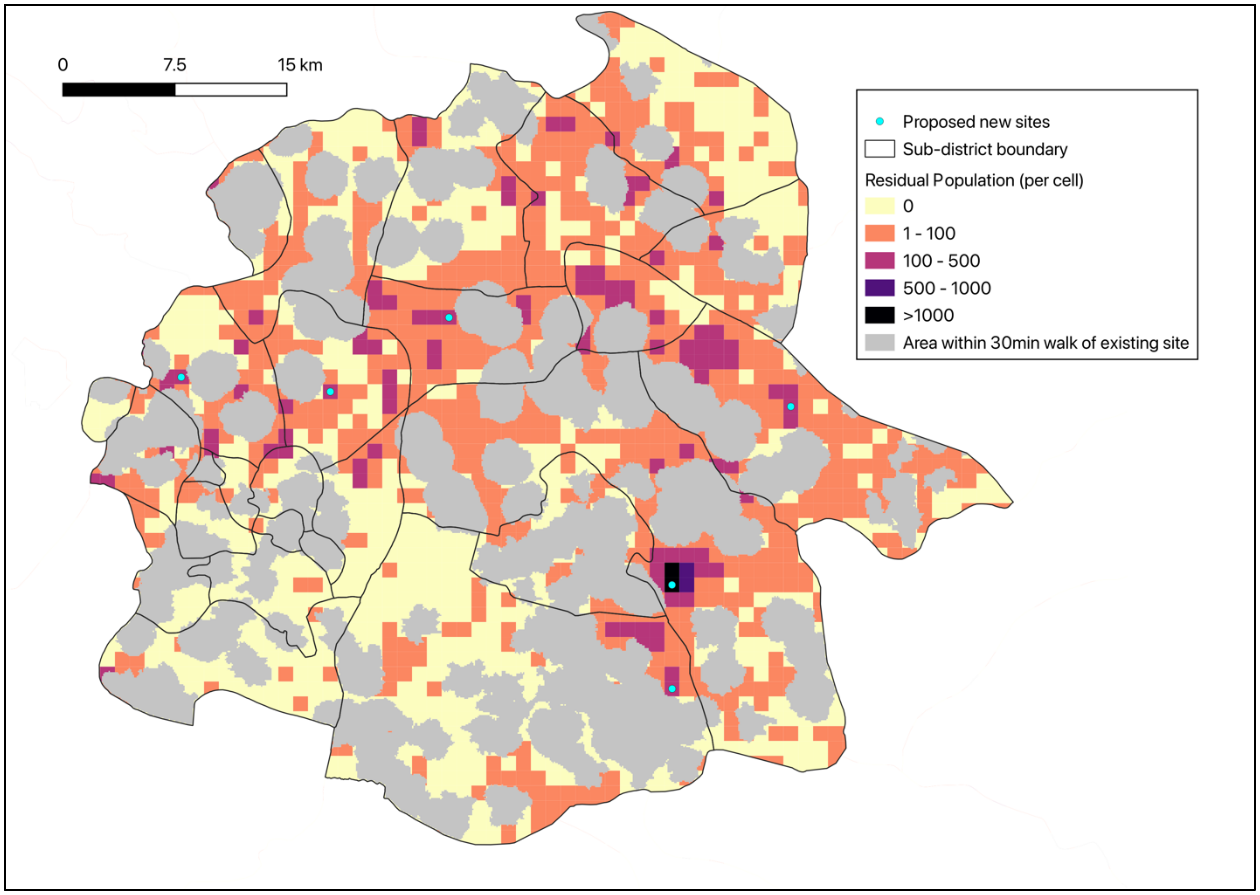

Supplementary Figure 4. High-resolution example of scale-up site selection in a subset of sub-districts in Ad Dali governorate. Gray areas represent locations already within 30-minute walk times of existing EPI services that are masked from the scale-up analysis. Colored squares represent the underlying residual population density (number of under-5 children beyond a 30-minute walk time of the nearest existing EPI site) within each 1km grid-cell. Blue circles represent proposed sites for new mobile clinics.

Mobile sites within 30-minute walk of fixed EPI site, by governorate			
Governorate	Mobile Sites (Total)	Mobile sites within 30-min walk time of nearest fixed vaccination site	
		Number	%
Ibb	617	142	23.0
Abyan	58	10	17.2
Sanaa City	11	7	63.6
Al Bayda	670	203	30.3
Taiz	129	64	49.6
Al Jawf	24	7	29.2
Hajjah	344	76	22.1
Al Hodeidah	22	3	13.6
Hadramawt	349	91	26.1
Dhamar	1295	424	32.7
Shabwah	1014	307	30.3
Sadah	92	22	23.9
Sanaa	480	103	21.5
Aden	5	5	100.0
Lahj	10	5	50.0
Marib	168	46	27.4
Al Mahwit	82	16	19.5
Al Maharah	22	3	13.6
Amran	354	140	39.5
Ad Dali	285	64	22.5
Raymah	62	15	24.2
Socotra	272	28	10.3
Total	6365	1781	28.0

Supplementary Table 4. Assessment of mobile site locations within 30-minute walk catchments of existing health facilities offering EPI.

Supplemental Table 5: Access to EPI services with fixed, mobile, and scale-up sites, using different grid-cells sizes for site selection							
Governorate	Total Under-5 Population	1km grid		3km grid		5km grid	
		No. children within 30 min-walk of EPI	% of Governorate Under-5 Population Covered	No. children within 30 min-walk of EPI	% of Governorate Under-5 Population Covered	No. children within 30 min-walk of EPI	% of Governorate Under-5 Population Covered
Ibb	448293	343318	76.6	339493	75.7	333157	74.3
Abyan	72069	45065	62.5	44681	62.0	44700	62.0
Sana a City	835434	787484	94.3	785150	94.0	775528	92.8
Al Bayda	121009	99239	82.0	99102	81.9	98934	81.8
Ta iz	483149	315000	65.2	312000	64.6	299698	62.0
Al Jawf	120037	80757	67.3	77720	64.7	68403	57.0
Hajjah	419576	327263	78.0	320250	76.3	304109	72.5
Al Hodeidah	607659	379423	62.4	376045	61.9	350984	57.8
Hadramawt	240086	176008	73.3	173325	72.2	166271	69.3
Dhamar	464031	344677	74.3	346611	74.7	338253	72.9
Shabwah	115179	87580	76.0	84138	73.0	84104	73.0
Sa dah	178765	110154	61.6	106810	59.7	95221	53.3
Sana a	178490	99667	55.8	97666	54.7	97512	54.6
Aden	179472	150727	84.0	148779	82.9	149339	83.2
Lahj	149306	81515	54.6	81623	54.7	80390	53.8
Ma rib	129950	82652	63.6	80410	61.9	80824	62.2
Al Mahwit	116862	79469	68.0	77644	66.4	76091	65.1
Al Maharah	42452	28202	66.4	27951	65.8	25168	59.3
Amran	224843	168885	75.1	164722	73.3	166067	73.9
Ad Dali	157272	115063	73.2	112031	71.2	107575	68.4
Raymah	97874	49234	50.3	49917	51.0	48451	49.5
Socotra	13339	12006	90.0	12006	90.0	11014	82.6
Total	5395148	3963390	73.5	3918073	72.6	3801792	70.5

Supplementary Table 5. Results of scale-up geographic accessibility analysis using 1km^2, 3km^2, and 5km^2 grids. Of the three variations, the 1km^2 grid selects locations that result in greaest gains in population coverage (number of under-5 children within a 30-minute walk of vaccination services).
