# Supplementary material for: Geospatial analysis and scale-up modelling of the impact of mobile programming on access to essential childhood vaccinations in Yemen
Source: Commun Med (Lond). 2025 Apr 18;5:126. doi: 10.1038/s43856-025-00762-5 (PMC12008410; doi:10.1038/s43856-025-00762-5)
Supplement: Supplementary file 2 — Description of Additional Supplementary Files [file 43856_2025_762_MOESM2_ESM.pdf]

Description of Additional Supplementary Files

File name: Supplementary Data 1
Description: Ranked list of proposed scale-up sites (GPS coordinates removed), by the residual population (number of under-5 children not within a 30-minute walk of an existing facility) within the 1km2 grid-cell for the new proposed site. Population-weighted time travel analyses were conducted to show the reduction in mean travel time (in minutes) and mean person travel time (in personminutes) to the nearest vaccination services for the corresponding grid cell.

File name: Supplementary Data 2
Description: Numerical results underlying Figure 3 in main text
